# Revealing the Nuclei Formation in Carbon-Inoculated Mg-3%Al Alloys Containing Trace Fe

**DOI:** 10.3390/ma12152478

**Published:** 2019-08-04

**Authors:** Chengbo Li, Shuqing Yang, Gan Luo, Hengbin Liao, Jun Du

**Affiliations:** School of Materials Science and Engineering, South China University of Technology, Guangzhou 510640, China

**Keywords:** grain refinement, carbon inoculation, thermodynamic model

## Abstract

In this study, Fe-bearing Mg-3%Al alloys were inoculated by combining carbon with or without Ca. Both processes can significantly refine the grain size of Mg-3%Al alloys. The highest refining efficiency can be obtained by carbon combined with Ca. The synergistic grain refining efficiency can be attributed to the constitutional undercooling produced by the addition of Ca. Two kinds of carbon-containing nuclei with duplex-phase particles and cluster particles were observed in the carbon-inoculated alloys. A thermodynamic model was established to disclose the formation mechanisms of the duplex-phase particles and Al_4_C_3_ cluster particles. This thermodynamic model is based on the change of Gibbs free energy for the formation of these two kinds of particles. The calculated results show that these two particles can form spontaneously, since the change of Gibbs free energy is negative. However, the Gibbs free change of the duplex-phase particle is more negative than the Al_4_C_3_ cluster particle. This indicates that the adsorption process is more spontaneous than the cluster process, and tiny Al_4_C_3_ particles are preferred to form duplex-phase particle, rather than gathering to form an Al_4_C_3_ cluster particle. In addition, the addition of Ca can reduce the interfacial energy between the Al_4_C_3_ phase and the Al–Fe phase and promote the formation of duplex-phase particles.

## 1. Introduction

For the past decade, grain refinement has become one of the most important routes to improve the mechanical properties of magnesium alloys [1,2,3]. The main grain refining methods for Al-bearing Mg alloys includes superheating [4], Elfinal processing [5], carbon inoculation [6,7,8], and solute addition [9,10,11,12,13]. Among these techniques, carbon inoculation is attracting widespread interest due to its low cost, low operating temperature, and less fading. To date, Al_4_C_3_, acting heterogeneously for α-Mg, has been a common mechanism of carbon inoculation. The amorphous carbon in the melt can react with liquid aluminium to form Al_4_C_3_, as revealed by Orbulov et al. [14]. In the process of inoculation, the bearing elements can significantly affect the inoculation and the microstructure of the metals [15,16]. Furthermore, the change of the material microstructure caused by the bearing elements is one of the factors leading to fatigue [17]. However, Al_4_C_3_ is very susceptible to trace impurity elements, such as Fe and Mn elements. One of the most significant discussions in carbon inoculated Mg–Al alloy is the effect of Fe on the grain refinement. Previous research findings on the role of Fe have been inconsistent and contradictory. Haitani et al. concluded that Fe has a negative impact on grain refinement because Fe tends to poison the potency of the Al_4_C_3_ nucleating particles by transforming them into Al-C-Fe-rich intermetallic compounds [18]. On the other hand, an experiment reported by Pan et al. demonstrated that Fe has a positive role on the reduction of grain size [19]. Our previous research has shown that Fe has no distinct effect on the grain refinement of a Mg–Al alloy by carbon inoculation according to the condition of Fe addition before carbon inoculation [20]. However, the existence of 0.1% Fe could accelerate the inoculant-fading, and the grain refining efficiency disappeared when the holding time was over 30 min [21]. Besides the effect on the nucleating particles, Fe also has a significant influence on mechanical and manufacturing processes [22,23].

Calcium is a commonly used element in gray cast iron [16]. It was found that Ca is an effective element to resist the phenomenon of inoculant-fading induced by Fe [24]. The addition of Ca was also proved to be not only inhibited the poisoning influence of Fe on grain refinement but also obtained further grain refining efficiency [25].

An interesting observation that has been found in the grain refinement of an Mg–Al alloy containing trace impurity elements—the alloy always exists duplex-phase structure particle. Numerous experiments have identified the existence of duplex-phase structure particle in the Mg–Al melt [26,27,28]. In a recent study conducted by Du et al., it was shown that Al–C-rich phases were coated on Al–Fe or Al–C–Fe-rich phases and that these duplex-phase particles could be potent substrates for α-Mg grains [6,26]. Similar to Fe, Mn is another impurity element that can influence grain refining efficiency. It is interesting to note that duplex-phase particles were also found in Mg–Al alloys containing Mn. For example, two kinds of duplex-phase particles of Al_4_C_3_ coated on Al_8_Mn_5_ and Al_0.89_Mn_1.11_ were found in AZ31 and AZ63 alloys refined by carbon-inoculation, respectively [28]. In addition, alkaline-earth elements, such Ca and Sr, can effectively promote the formation of duplex-phase particles [6,26]. Furthermore, it has been proven that further grain refining efficiency could be obtained for the Mg–Al alloys modified by combining the addition of Ca or Sr with carbon inoculation due to the synergistic action [25].

Considering all of this evidence, it seems that duplex-phase particles appearing to refine magnesium alloys containing impurity elements is a widespread phenomenon. However, up to now, no studies have yet investigated the formation mechanism of the duplex-phase particle. The relationship between Ca and the duplex-phase particle is still unclear. A thermodynamic model is an effective way to describe this formation tendency [29]. In this study, a thermodynamic model will be established to explain the formation mechanism of the duplex-phase particle. The effect of Ca on the formation of the duplex-phase particle will also be discussed in detail.

## 2. Materials and Methods

Mg-3%Al was chosen as the base alloy in the present study. The base alloy was produced from relatively high purity magnesium (99.98%) and high purity aluminum (99.99%). The base metals of Mg and Al were provided by Yinguang Magnesium Industry (Group) Co., Ltd. (Yuncheng, China) and Zhongnuo advanced material Co., Ltd., (Beijing, China), respectively. The alloys were melted with an MgO crucible in an electric resistance furnace at a temperature of 760 °C. The melt was covered by a protective mixed gas (SF_6_ and N_2_). In all melting operations, melts were stirred for 1 min and then cast into a tapered iron mold with a size of φ20 × φ22 × 30 mm^3^, which was preheated at 500 °C.

Al-15%Fe and Mg-10%Ca were used as master alloys for Ca and Fe additions. The carbon pellets were made from Mg, Al, and graphite powders with a mass radio of 4:5:1. The mix powders were poured into the mold and then mixed mechanically. Using a cold isostatic press (CIP) pressed at a pressure of 130 MPa for 180 s, carbon pellets with diameters of 30 mm were finally produced. To exactly control the Al content in the Mg–Al melt, the amounts of Al in the pellets and the Al-15%Fe master alloy were carefully taken into consideration. The melting process used in this study is similar to that in our previous research [6]. The added contents of Fe, carbon, and Ca were 0.05%, 0.2%, and 0.2% (mass ratio, the same below) of the melt, respectively.

Our samples were prepared according to the procedure used by Du [8,30]. Metallographic samples were cut in the horizontal direction at the position of 15 mm from the bottom of the samples. In order to observe the grain microstructures, the sample was divided into two parts. One part of the sample was heat treated at 420 °C for 6 h and then cooled in the air. These specimens were polished and subsequently chemically etched. The etchant consists of picric acid (4.2 g), glacial acetic acid (10 mL), ethyl alcohol (70 mL), and distilled water (10 mL). The grain microstructures were observed using the Leica DFC320 type optical microscope (Wetzlar, Germany). The linear intercept method described in the American Society for Testing Materials (ASTM) standard E112-88 was used to evaluate the grain size. The as-cast samples were etched by 2 vol.% nitride acid ethanol solution and subsequently observed by electron probe microanalyzer (EPMA-1600, Shimadzu, Kyoto, Japan) equipped with energy dispersive X-ray spectroscopy (EDAX) and a wavelength dispersion spectrometer (WDS). In order to exactly identify the single phase particle, a transmission electron microscope (TEM, JEM-2100, JEOL, Tokyo, Japan) was used in this study.

## 3. Results

### 3.1. Grain Refining Efficiency

**Figure 1:**
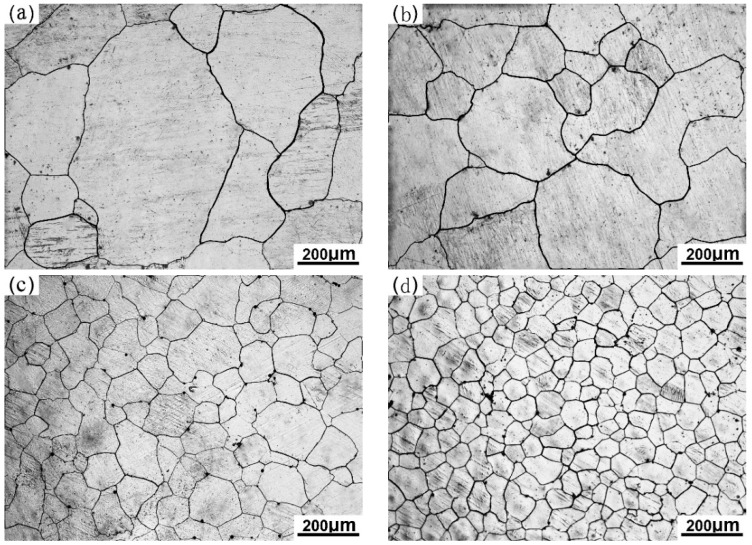
Grain morphologies of (**a**) the original Mg-3%Al alloy, (**b**) the Mg-3%Al alloy containing 0.05%Fe, (**c**) the Mg-3%Al alloy containing 0.05%Fe refined by carbon inoculation, and (**d**) the Mg-3%Al alloy containing 0.05%Fe refined by carbon inoculation, combined with 0.2%Ca.

Figure 1 shows the grain microstructures of the Mg-3%Al alloy treated with different processes. Coarse grains are observed in the Mg-3%Al alloy sample without any treatment. The grain size of the original Mg-3%Al alloy is about 632 ± 15 μm, as shown in Figure 1a. It can be seen from Figure 1b that the grain size of the Mg-3%Al alloy containing 0.05%Fe is 425 ± 26 μm. As shown in Figure 1c, the grain size of the Mg-3%Al alloy containing 0.05%Fe is significantly refined by carbon inoculation. Its grain size is decreased to about 185 ± 6 μm. Mg-3%Al alloy containing 0.05%Fe is further refined by carbon inoculation, combining with the 0.2%Ca addition, as shown in Figure 1d. The grain size clearly decreased from 185 ± 6 μm to 115 ± 5 μm.

### 3.2. Observation of Nucleating Particles

**Figure 2:**
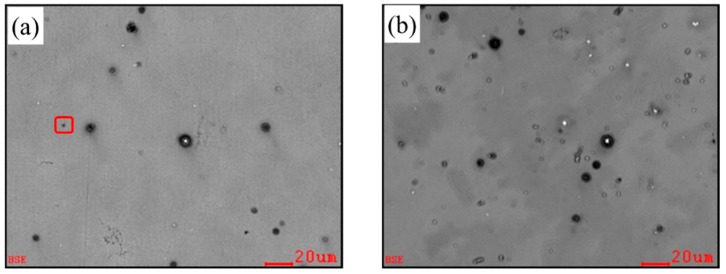
Electron probe microanalyzer (EPMA)-back-scattered electron (BSE) micrographs of the 0.05%Fe containing Mg-3%Al alloy refined by carbon inoculation (**a**) and carbon inoculation combined with the addition of 0.2%Ca (**b**).

In order to investigate the nucleation particles, we chose Fe containing Mg-3%Al alloy treated by carbon inoculated and carbon combined with 0.2%Ca for characterization. The images characterized by electron probe microanalyzer (EPMA) back-scattered electron (BSE) are shown in Figure 2a,b, respectively.

Two typical particles are found in both samples: one is a particular duplex-phase particle with a white core surrounded by grey halos; the other is a tiny gray particle. The diameter of the duplex-phase particle is about 3–5 μm, and the gray particle is about 1–2 μm. Compared with the sample treated only by carbon inoculation, there were many white particles visible in the sample of the carbon with added Ca, as shown in Figure 2b.

**Figure 3:**
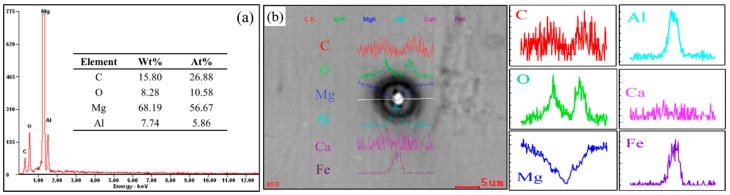
EPMA analyses of two typical particles: (**a**) point analysis of the single particle; (**b**) line analysis of the duplex-phase particle.

The chemical compositions of these two typical particles were characterized by EPMA point and line analyses, respectively. Figure 3a shows the EPMA point analysis of the single particle in Figure 2a (the area denoted by the red line. As shown in Figure 3a, there were three obvious peaks for the Al, C, and O elements. Unlike the duplex-phase particles, this single phase particle does not contain the Fe element. These single phase Al–C–O particles are formed in situ by Al and C elements in the melt. It can be inferred that the particles are actually Al_4_C_3_ particles. The element of O could be the result of contamination during sample preparation, via the reactions ${\mathrm{Al}}_{4}C_{3}{+H}_{2}O\;\to {\;\mathrm{Al}(\mathrm{OH})}_{3}{+\mathrm{CH}}_{4}↑$ [8,28,31]. This evidence suggests that the single phase particle could be formed by tiny Al_4_C_3_ particle clusters.

It can clearly be seen from Figure 3b that the contents of Al and Fe in the core are higher than those in the grey halos and the Mg matrix. At the edge of the gray halo, there exist two obvious peaks of carbon and oxygen, respectively. It can be reasonably inferred that the duplex-phase particle is constitute by an Al–C layer coated on the Al–Fe core. The Al–C layer is actually formed by Al_4_C_3_ particles. Similar duplex-phase particles are easily observed in our previous studies [26,27].

## 4. Discussion

### 4.1. Grain Refinement Mechanism

**Figure 4:**
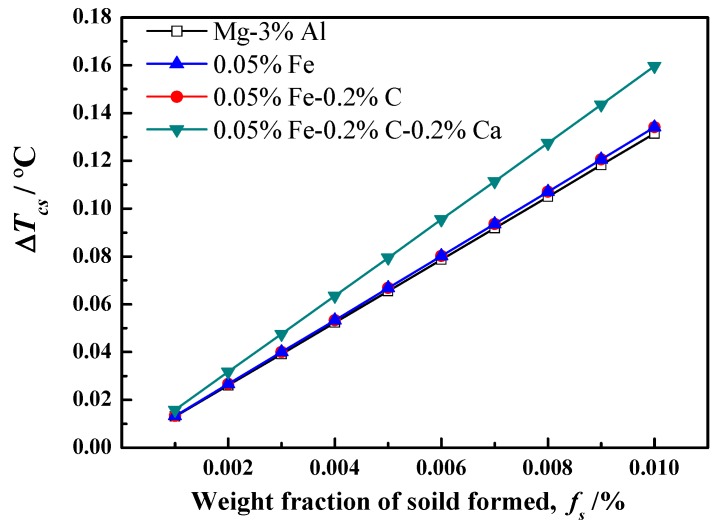
The totally constitutional undercooling $\Delta T_{cs}$ produced at the solid–liquid interface during the solidification.

In Figure 1, there is clearly a trend of grain size decreasing by different treated processes. After the addition of 0.05%Fe, the grain size of Mg-3%Al decreases slightly. This means that the constitutional undercooling produced by the Fe element cannot be neglected, especially as Fe has a very high growth restriction factor (GRF = 52.56) [20]. Therefore, the effect of constitutional undercooling resulting from Fe was determined in this study. The total constitutional undercooling ($\Delta T_{CS}$) produced at the solid–liquid interface can be calculated by the following equation:

(1) $$\Delta T_{CS}=m_{l}c_{0}\left(1-\frac{1}{{\left(1-f_{s}\right)}^{P}}\right)$$

where *f_S_* is the solid mass fraction, $m_{l}$ is the slope of the liquids, $c_{0}$ is the alloy composition, and *P* = 1 − *k*, *k* is the equilibrium distribution coefficient. The calculation results are shown in Figure 4, and the relevant parameters are listed in Table 1. As shown in Figure 4, 0.05%Fe addition can improve the constitutional undercooling, but the increase is limited. As an inevitable impurity element, the content of Fe in Mg alloy is very low. Even in commercial raw materials, the content of Fe is less than 0.05%. The solubility of carbon in the Mg–Al melt is too small. It has been reported that the solubility of carbon in Mg-3%Al and Mg-9%Al is about 20 ppm [32]. Therefore, the constitutional undercooling produce by carbon was not taken into calculation, and the curve of carbon addition overlapped with addition of Fe, as shown in Figure 4. It is worth noting that the constitutional undercooling clearly increased after adding 0.2%Ca. In this research, Ca was added in the Mg-3%Al melt by Mg-10%Ca master alloys. Mg-10%Ca alloys are decomposed completely and dissolved into the melt as a solute, since the solubility of Ca in the Mg melt is about 0.8% at 760 °C [33]. It is known that Ca has a strong tendency for segregation. Therefore, the solute of Ca could greatly affect the constitutional undercooling and, consequently, restrict grain growth during the solidification process. The results in Figure 1d also confirm that the grain size is further refined after Ca addition. 

The constitutional undercooling shows little change, while the grain size is significantly refined after carbon inoculation, as shown in Figure 1c and Figure 4. Therefore, the significant grain refinement after carbon inoculation cannot be explained by the constitutional undercooling. The effect of nucleating particles on grain size must be considered. In this study, there mainly exist two kinds of effective nucleation particles. One is the Al_4_C_3_ cluster particle, and the other is the duplex-phase particles. The interfacial phases between the Mg melt and these two heterogeneous nucleation particles are both Al_4_C_3_, which is believed to be a potent nucleating substrate for primary α-Mg by crystallography calculation [35]. However, the different diameters of these two particles results in different refining efficiencies. The diameter of the nucleating particle is a very important factor for nucleation efficiency. Greer et al. put forward a model establishing the relationship between nucleation particle size and nucleation undercooling ($\Delta T_{n}$) [36], as shown in Equation (2):

(2) $$\Delta T_{n}=\frac{4\sigma_{SL}}{\Delta s_{v}d_{p}}$$

where $\sigma_{SL}$ is the solid-liquid interface energy, $\Delta s_{v}$ is the entropy of fusion per unit volume, and $d_{p}$ is the diameter of the nucleating particle. According to Equation (2), the lager the particle size is, the smaller the nucleation undercooling $\Delta T_{n}$ is required. Large particles have a higher potency to act as heterogeneous nucleation sites. As shown in Figure 2, The diameters of duplex-phase particle are about 3–5 μm, while the diameters of Al_4_C_3_ cluster particle are about 1–2 μm. Deduced from this model, the grain refinement efficiency of the duplex-phase particle is higher than that of the Al_4_C_3_ cluster particle.

### 4.2. Formation Process of Duplex-Phase and Al_4_C_3_ Cluster Particles

**Figure 5:**
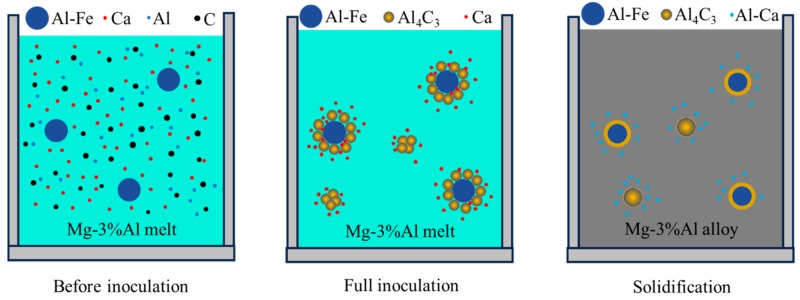
Schematic diagrams of the formation process of the duplex-phase and Al_4_C_3_ cluster particles.

A brief description of the formation process of nucleating particles is given in Figure 5. In the initial stage of inoculation, Al–Fe particle Ca solutes exist in the Mg-3%Al melt. In the process of inoculation, Al_4_C_3_ can be formed by the following reaction [8]:

(3) $${3C\;+\;4\mathrm{Al}\;=\;\mathrm{Al}}_{4}C_{3},\;\Delta G_{1033}\;=\;-30.9\;\mathrm{KJ}\cdot {\mathrm{mol}}^{-1}$$

These thermodynamic data indicate that the formation of Al_4_C_3_ is thermodynamically possible at a temperature of 760 °C. After full inoculation, some of Al_4_C_3_ particles are adsorbed onto the surface of Al–Fe particles to form duplex-phase particles. The other Al_4_C_3_ particles agglomerate to form Al_4_C_3_ cluster particles. During the inoculation, Ca segregates towards to the interface of the Al–Fe and Al_4_C_3_ particles, as shown in Figure 5. Finally, Ca reacts with Al to form Al–Ca particles during solidification.

**Figure 6:**
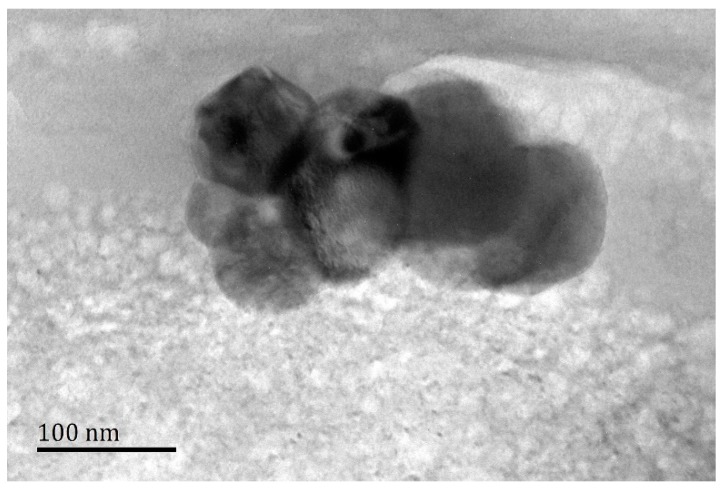
The TEM microstructure of the Al_4_C_3_ cluster from several tiny particles.

The exactly microstructure of Al_4_C_3_ cluster particles are confirmed by TEM as shown in Figure 6. As can be seen from the TEM image, the diameter of in-situ formation Al_4_C_3_ particles is very small (nano scale). These tiny particles have a high surface energy and are easily to agglomerate. Therefore, the single phase Al_4_C_3_ particle is constituted by a cluster of tiny Al_4_C_3_ particles.

**Figure 7:**
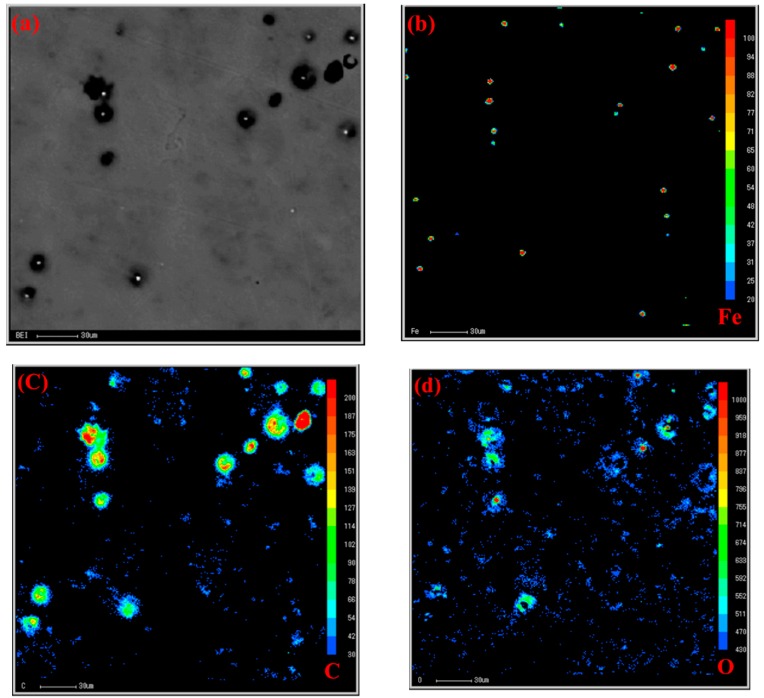

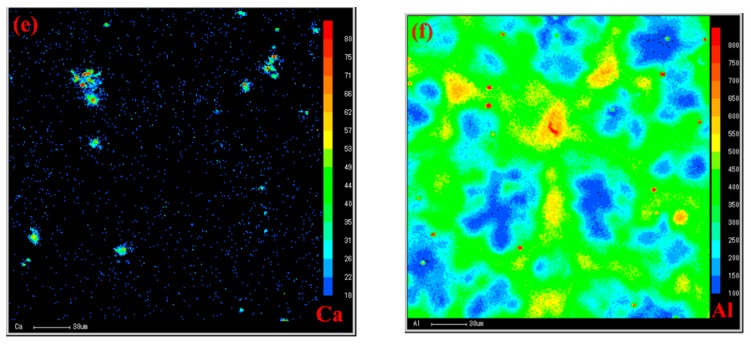
Electron probe microanalyzer with a wavelength dispersion spectrometer (EPMA-WDS) map analysis of Mg-3%Al-0.05%Fe refined by carbon combining with Ca inoculation, with a holding time of 80 min. (**a**) The region of EPMA-WDS map analysis; (**b**–**f**) the distribution of the Fe, C, O, Ca, and Al elements, respectively.

To reveal the relationship between the role of Ca and the duplex-phase particles, an electron probe microanalyzer with a wavelength dispersion spectrometer (EPMA-WDS) map analysis was carried out to identify the element distribution, as shown in Figure 7. It can be seen from Figure 7a that there are three typical duplex-phase structure particles and many Al_4_C_3_ cluster particles. Fe appears at the center of the duplex-phase particles. High concentrations of C and O were found to surround the Fe element and the distribution of these two elements always overlaps. Interestingly, both Al_4_C_3_ cluster particles and duplex-phase particles were surround by Ca. Unlike the distribution of the C and O elements, the Ca element did not form a complete shell coating on the core of Al–Fe or the Al_4_C_3_ cluster particles. Figure 7e clearly shows a trend where the Ca element is segregated towards the duplex-phase and Al_4_C_3_ cluster particles.

### 4.3. Establishment of the Thermodynamic Model

In the author’s previous experiment [37], the amount of tiny Al_4_C_3_ particles with sizes ranging from 10 to 1000 nm could be found in the melt. Based on the discussion above, these sub-micron particles could be extremely easy to adsorb on relatively large Al–Fe rich particles or cluster together due to their high surface energy. Classical thermodynamic theory can be used to reveal the formation mechanism of two kinds of particles by a change in Gibbs free energy. The Gibbs free energy of the system associated with the formation of a new particle (duplex-phase or Al_4_C_3_ cluster particles) can be expressed as

(4) $$\Delta G=\sum S_{i}\times \gamma_{i}-\sum S_{j}\times \gamma_{j}$$

where *S* is the total surface area per unit mole of particles (m^2^/mol) and *γ* is the interfacial energies at the boundaries (J/m^2^). The element of *i* and *j* represent the particles in the melt after and before the formation of new particles, respectively.

**Figure 8:**
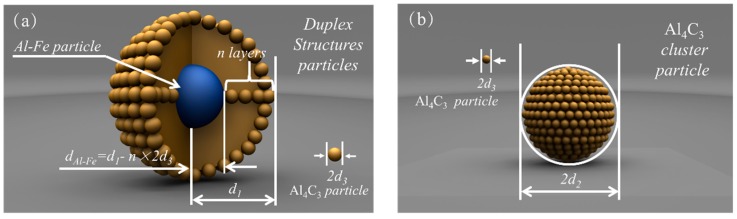
Calculation model of the duplex and single structure particle.

In order to calculate the change in the Gibbs free energy of the system associated with the formation of adsorption and clustering, the assumptions employed are as follows:

(1) All the duplex-phase particles and Al_4_C_3_ cluster particles are spherical, and their radii are represented by *d*_1_ and *d*_2_, respectively, as shown in Figure 8a,b.

(2) The radius of all tiny Al_4_C_3_ particles is denoted by *d*_3_.

(3) Al–Fe particles are completely coated by tiny Al_4_C_3_ particles. The thickness of one layer is approximate to a tiny Al_4_C_3_ particle’s radius. The number of adsorption layers is represented by *n*. Therefore, the radius of the Al–Fe particle is *d*_Al-Fe_ = *d*_1_ – *n* × 2*d*_3_.

(4) The tiny Al_4_C_3_ particles are distributed uniformly in the Mg–Al melt before adsorbing to the Al–Fe particle or clustering to a relatively large Al_4_C_3_ particle. 

(5) The total number of the tiny Al_4_C_3_ particles in the duplex-phase particle and the Al_4_C_3_ cluster particle is the same.

For a duplex-phased structure particle, the volume of the outer Al_4_C_3_ layer ($V^{\prime }$) can be expressed as

(5) $$V^{\prime }=\frac{4}{3}\pi \;\times \;\left(d_{1}^{3}\;-d_{Al-Fe}^{3}\right)=\;\frac{4}{3}\pi \;\times \;(d_{1}^{3}\;-\;\left(d_{1}\;-\;n\;\times \;2d_{2}{)}^{3}\right)$$

The total number of the tiny Al_4_C_3_ particles (N) that adsorbed on the surface of the Al–Fe rich particle can be given as

(6) $$N\;=\;\frac{V^{\prime }}{\frac{4}{3}\;\times \;\pi d_{3}^{3}}\;=\;\frac{d_{1}^{3}{\;-\;(d}_{1}{-\;n\;\times \;2d}_{3}{)}^{3}}{d_{3}^{3}}.$$

It is assumed that after carbon containing pellets were plunged into the melt, the total number of tiny Al_4_C_3_ particles (N) and one Al–Fe particle were uniformly distributed in the melt. In the process of carbon inoculation, tiny Al_4_C_3_ particles are completely adsorbed onto the surface of Al–Fe particles to form a duplex-phase particle. According to Equation (4), the change in the Gibbs free energy ($\Delta G_{duplex}$) of the system associated with the formation of the duplex-phase particle shown in Figure 8a can be written as

(7) $$\Delta G_{dupex}=S_{dupex}\times \gamma_{{\mathrm{Al}}_{4}C_{3}/\mathrm{Mg}}+S_{\mathrm{Al}-\mathrm{Fe}}\times \gamma_{{\mathrm{Al}}_{4}C_{3}/\mathrm{Al}-\mathrm{Fe}}-\left(S_{\mathrm{Al}-\mathrm{Fe}}\times \gamma_{\frac{\mathrm{Al}-\mathrm{Fe}}{\mathrm{Mg}}}+\;S_{N{-\mathrm{Al}}_{4}C_{3}}\times \gamma_{{\mathrm{Al}}_{4}C_{3}/\mathrm{Mg}}\right)$$

where the $S_{duplex}$ is the surface area of duplex-phase particle and the $\gamma_{{\mathrm{Al}}_{4}C_{3}/\mathrm{Al}-\mathrm{Fe}}$ is the interfacial energy between Al_4_C_3_ phase and Mg melt. $S_{\mathrm{Al}-\mathrm{Fe}}$ is the surface area of the Al–Fe particle and $\gamma_{{\mathrm{Al}}_{4}C_{3}/\mathrm{Al}-\mathrm{Fe}}$ is the interfacial energy between the Al_4_C_3_ phase and the Al–Fe phase, since the Al–Fe particle is surround by tiny Al_4_C_3_ particles. Before carbon inoculation, tiny Al_4_C_3_ particles (N) and one Al–Fe particle were uniformly distributed in the melt. Therefore, $\gamma_{\mathrm{Al}-\mathrm{Fe}/\mathrm{Mg}}$ is the interfacial energy between Al–Fe phase and the Mg melt. $\;S_{N{-\mathrm{Al}}_{4}C_{3}}$ is the surface area of the all the tiny Al_4_C_3_ particles. The $S_{duplex}$, $S_{\mathrm{Al}-\mathrm{Fe}}$, and $\;\;S_{N{-\mathrm{Al}}_{4}C_{3}}$ can be represented by the radii of *d*_1_, *d*_2,_ and *d*_3_ via the following function:

(8) $$S_{duplex}=4\pi d_{1}^{2}$$

(9) $$S_{\mathrm{Al}-\mathrm{Fe}}=4\pi {\left(d_{1}-n\times 2d_{3}\right)}^{2}$$

(10) $$S_{N{-\mathrm{Al}}_{4}C_{3}}=4\pi d_{3}^{2}\times N=\;4\pi d_{3}^{2}\times \frac{d_{1}^{3}-{\left(d_{1}-n\times 2d_{3}\right)}^{3}}{d_{3}^{3}}.$$

Finally, the $\Delta G_{duplex}$ be represented by the radius of *d*_1_ and *d*_2_ via the following function:

(11) $$\begin{array}{c} \Delta G_{duplex}=4\pi d_{1}^{2}\times \gamma_{{\mathrm{Al}}_{4}C_{3}/\mathrm{Mg}}+4\pi {\left(d_{1}-n\times 2d_{3}\right)}^{2}\times \gamma_{\frac{{\mathrm{Al}}_{4}C_{3}}{\mathrm{Al}-\mathrm{Fe}}}\\ -(4\pi \left(d_{1}-n\times 2d_{3}{)}^{2}\times \gamma_{\frac{\mathrm{Al}-\mathrm{Fe}}{\mathrm{Mg}}}+4\pi d_{3}^{2}\times \frac{d_{1}^{3}-{\left(d_{1}-n\times 2d_{3}\right)}^{3}}{d_{3}^{3}}\times \gamma_{{\mathrm{Al}}_{4}C_{3}/\mathrm{Mg}}\right). \end{array}$$

The Gibbs free energy change of the cluster particles ($\Delta G_{cluster}$) can also be calculated using the same model. The $\Delta G_{cluster}$ can be represented by the following function:

(12) $$\Delta G_{cluster}=\gamma_{{\mathrm{Al}}_{4}C_{3}/\mathrm{Mg}}\times \left(S_{cluster}-S_{N{-\mathrm{Al}}_{4}C_{3}}\right)=\;\gamma_{{\mathrm{Al}}_{4}C_{3}/\mathrm{Mg}}\times 4\pi \times \left(d_{2}^{2}-d_{3}^{2}\times \frac{d_{1}^{3}-{\left(d_{1}-n\times 2d_{3}\right)}^{3}}{d_{3}^{3}}\right)$$

Theoretically, the interfacial free energy at the nucleating interface is believed to be a key factor in controlling heterogeneous nucleation efficiency. However, the interfacial energy is usually difficult directly measure for solid–liquid or solid–solid interfaces. As the crystal planes are usually bound with the lowest interface energy the Al_4_C_3_/Al–Fe interface, it can be regarded as a coherent interface. Therefore, the interface energy $\gamma_{{\mathrm{Al}}_{4}C_{3}/\mathrm{Al}-\mathrm{Fe}}$ could be taken as 0.1 J/m^2^, since the coherent interface energy is generally considered to be in the range from 0 to 0.2 J/m^2^ [38].

The interfacial energy between the two phases can be estimated by Girifalco–Good’s [38] equation:

(13) $$\delta_{A/B}=\tau_{A}+\tau_{B}-2\phi_{AB}{\left(\tau_{A}+\tau_{B}\right)}^{1/2}$$

where *δ_A/B_* is the interfacial energy between the A phase and B phase, *τ_A_* and *τ_B_* are the surface energy of A phase and B phase respectively, and *ϕ_AB_* is the interaction between these two phases which typically ranges around 1.

Pan calculated the surface energy of the FeAl (110) alloy surface by first-principles calculations [39]. The result shows that the Fe:Al = 1:1, Fe:Al = 1:2, and Fe:Al = 1:3 surface structures are stable, and the surface energy of these three surface structures ranges from 1.28 J/m^2^ to 2.4 J/m^2^. In our previous study, AlFe_3_ was considered as a possible compound with a surface energy *τ*_Al–Fe_ of 2.24 J/m^2^ [24]. Li analysed slabs of Al_4_C_3_ (0001) by first-principles calculations [40]. The calculation shows that the Al-terminated surface is more stable than the C-terminated surface, and the surface energy of C-termination surface is about 2.7 J/m^2^, which could be taken as the surface energy of the Al_4_C_3_ phase. Substituting *τ*_Al–Fe_ = 2.24 J/m^2^, *τ*_Al–C_ = 1.33 J/m^2^, and *τ*_Mg(*l*)_ *=* 0.577 J/m^2^ into Girifalco–Good’s equation (Equation (13)), the interfacial energy of $\gamma_{{\mathrm{Al}}_{4}C_{3}/\mathrm{Mg}}$ and $\gamma_{\mathrm{Al}-\mathrm{Fe}/\mathrm{Mg}}$ can be calculated as 0.16 J/m^2^ and 0.56 J/m^2^, respectively. 

**Figure 9:**
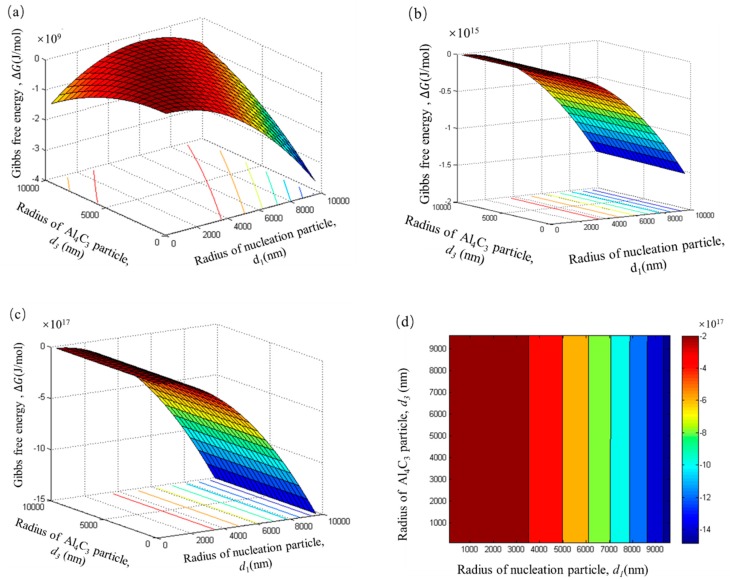
The Gibbs free energy of the duplex phase particle with different adsorption layers. (**a**) n = 1, (**b**) n = 100, (**c**) n = 1000, (**d**) the contour map of the n = 1000.

**Figure 10:**
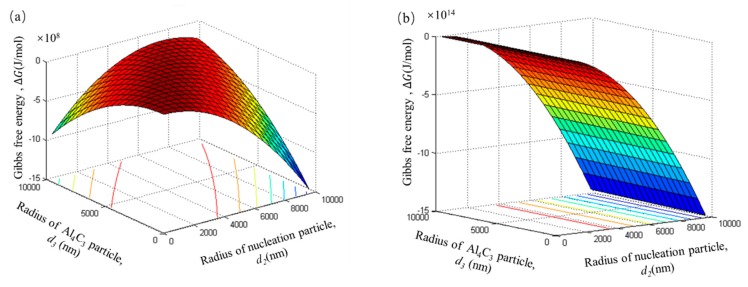

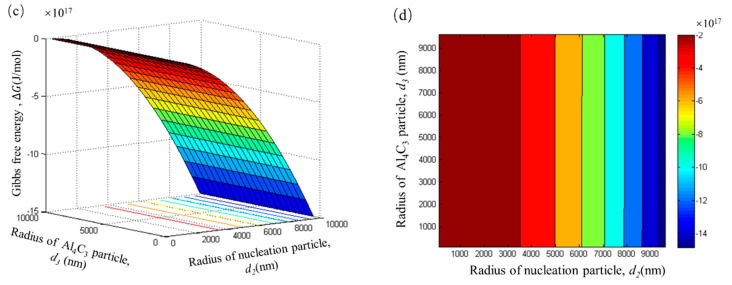
The Gibbs free energy of the Al_4_C_3_ cluster particles with different number tiny Al_4_C_3_ particles. The number of tiny Al_4_C_3_ particles is calculated by Equation (6). (**a**) n = 1, (**b**) n = 100, (**c**) n = 1000, (**d**) the contour map of n = 1000.

For ease of comparison, the radii of the duplex-phase particle and Al_4_C_3_ cluster particles were taken as the radii of the nucleation particles in Figure 9 and Figure 10. In order to established a thermodynamics model close to the real experimental process, the radii of the duplex-phase particle(*d*_1_), the Al_4_C_3_ cluster particle (*d*_2_), and the tiny Al_4_C_3_ (*d*_3_) particle range from 0 to 10 μm.

### 4.4. Calculation Results from Thermodynamic Model

As shown in Figure 9a, all the Gibbs free energy within the range of *d*_1_ and *d*_3_ is negative, when Al–Fe particles only adsorb a single layer of Al_4_C_3_ particles. The Gibbs free energy is negative to about a 10^9^ J/mol magnitude. This result indicates that the Al–Fe particle adsorbing tiny Al_4_C_3_ particles to form a duplex-phase particle is a spontaneous process in the initial stage of carbon inoculation. When the number of adsorbed layers increases from 100 to 1000, the Gibbs free energy of the system is more negative, and the order of magnitude increased from 10^15^ to 10^17^, respectively. These results suggest that the growth of the duplex-phase particle by the Al–Fe particle adsorbing the tiny Al_4_C_3_ particle is also a spontaneous process. As Figure 9b,c shows, an increase in the absorption of the layer produced no significant change in the shape of the pattern. In order to predict the trend of Gibbs free energy, which changes alongside the nucleated particles’ radii, the adsorption date of 1000 layers is selected to draw a contour map, as shown in Figure 9d. The contour map is the region in the same colour expressing equal Gibbs free energy.

It can be seen from Figure 9d that the Gibbs free energy is significantly reduced, while the radius of the nucleation particles increases. This can be attributed to the larger particles having a larger surface area that can absorb more tiny Al_4_C_3_ particles. Therefore, the total surface area reduces and makes the Gibbs free energy of the system more negative. It should be noted that the Gibbs free energy has nothing to do with the radius of tiny Al_4_C_3_ particles.

In this thermodynamics model, it is assumed that the number of tiny Al_4_C_3_ particles in the Al_4_C_3_ cluster particle is equal to the adsorption layer of duplex-phase particle. The total number of tiny Al_4_C_3_ particle can be calculated by Equation (6). In the initial stage, the Gibbs free energy change of the Al_4_C_3_ cluster particle also shows a high negative value of 10^8^ J/mol. This indicates that Al_4_C_3_ cluster particles also have a tendency to form spontaneously. With an increase in the number of tiny Al_4_C_3_ particles, this spontaneous trend becomes more and more obvious, as shown in Figure 10b,c. From the decreasing trend of Gibbs free energy (Figure 10d), it can be seen that the Al_4_C_3_ cluster particle can grow via tiny Al_4_C_3_ particle clusters.

From the above experimental results and thermodynamic model analysis, it can be concluded that the two kinds of particles coexist and have a competitive relationship. The data of adsorbing 1, 100, and 1000 layers were used to investigate the competition relationship between these two types particles at different stages of carbon inoculation. The competitive trend between these two types particles can be compared by reducing the Gibbs free energy of the system. The Gibbs free energy of the competitive trend $\Delta G_{competitive}$ can be expressed by

(14) $$\Delta G_{competitive}=\Delta G_{duplex}-\Delta G_{cluster}.$$

In order to compare the formation trend of duplex-phases and Al_4_C_3_ cluster particles at different radius, the radii of both particles are expressed by *d*:

(15) $$d=\{\begin{array}{c} d_{1},\;\;\Delta G_{competitive}<0\\ d_{2},\;\;\Delta G_{competitive}>0 \end{array}.$$

As the relationship of Equation (15) shows, if the $\Delta G_{competitive}$ is less than 0, the tendency of duplex-structure particle formation is more obvious. Otherwise, the tendency to form Al_4_C_3_ cluster particles is more obvious.

**Figure 11:**
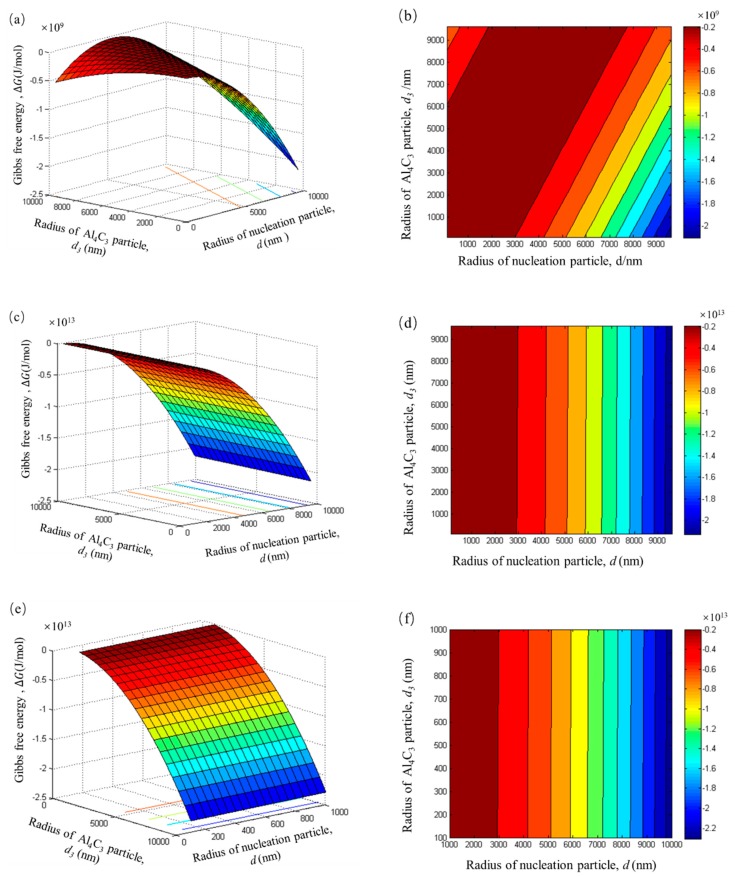
The Gibbs free energy of the competitive trend with different layers. (**a**,**b**): n = 1, (**c**,**d**): n = 100, (**e**,**f**): n = 1000.

The result calculated by Equation (14) is shown in Figure 11. As can been from Figure 11a, the Gibbs free energy is always negative. It can be deduced that the adsorption process is more spontaneous than the cluster process and the tiny Al_4_C_3_ particles prefer to form duplex phase particles rather than gather to form Al_4_C_3_ cluster particles. However, in the range of a specific particle radius, the $\Delta G_{competitive}$ is close to zero, as shown in Figure 11a (the dark red region). These results suggest that within a certain particle radius, the changes of Gibbs free energy by these two types of particles are almost equal. Thus, if the particle size is within this range, the trend of formation for the two types of particles is equal. This particle size range is accurately reflected in the dark red region of the contour map in Figure 11b. It is interesting to note that when the layers increased, there is a clear trend showing a decrease of the dark red region area, as shown in Figure 11d,f. These results indicate that the trend of forming stable duplex-phase particles becomes more and more obvious during the carbon inoculation process. This result is also confirmed by our previous research, which showed that the duplex phase particles kept stable when prolonging the holding time to 80 min and exhibit significant fading resistance [6].

### 4.5. The Role of Ca Solute in Carbon Inoculated Mg-3%Al Alloy

As discussed above, the solute of Ca can provide constitutional undercooling in front of the nucleus/liquid. Furthermore, Ca is a surface active element that can reduce the interfacial energy effectively between the two phases by segregating around the interface of the two phases. As shown in Figure 7e, the solute of Ca is segregated around the duplex-phase. It can be inferred that the addition of Ca reduces the interfacial energy between the Al_4_C_3_ phase and the Al–Fe phase. The change in Gibbs free energy can be used to describe the effect of adding Ca on the formation of the duplex phase particles. The change of the Gibbs free energy after Ca addition can be expressed by the equation:

(16) $$\Delta G_{change}=\Delta G_{duplex-Ca}-\Delta G_{duplex}.$$

**Figure 12:**
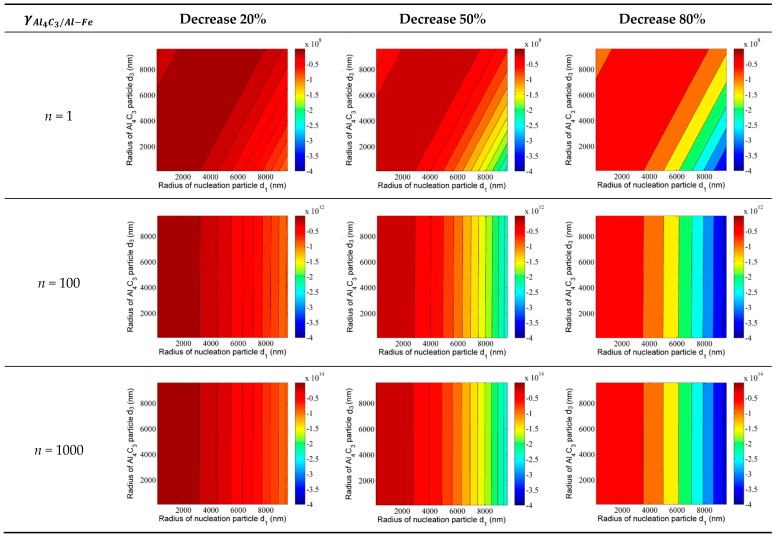
The change of Gibbs free energy after Ca addition. The number of adsorption layers is 10, 100, and 1000, respectively.

Unfortunately, there are no accurate data on the reduction of interfacial energy between the Al_4_C_3_ phase and Al–Fe phase after Ca addition. Therefore, we assume that the interfacial energy $\gamma_{{\mathrm{Al}}_{4}C_{3}/\mathrm{Al}-\mathrm{Fe}}$ can be reduced by 20%, 50%, and 80%. The changes in the Gibbs free energy after Ca addition are shown in Figure 12. After adding Ca, all changes in Gibbs free energy with different adsorption Al_4_C_3_ layers are negative. Furthermore, with the change of Gibbs free energy, the energy becomes more negative with a decrease in the interfacial energy $\gamma_{{\mathrm{Al}}_{4}C_{3}/\mathrm{Al}-\mathrm{Fe}}$. As shown in Figure 12, the color of the contour map changes from dark red to dark blue. This indicates that the addition of Ca can reduce the resistance of forming duplex-phase particles. In other words, the reduction interface energy induced by Ca would promote the formation of duplex phase particles. As the number of adsorption layers increases to 100 and 1000 layers, the trend in Gibbs free energy changes remains unchanged. Furthermore, the orders of magnitude are negative (from 10^12^ to 10^14^ J/mol), with the adsorption layers increasing from 100 to 1000 layers. This indicates that the size of the duplex phase particle could increase by adsorbing more Al_4_C_3_ layers after Ca addition. Based on the discussion above, the duplex-phase particles have a higher refinement efficiency due to the larger size of the duplex-phase particle. Therefore, the grain refining efficiency can be further improved by the addition of Ca.

## 5. Conclusions

1. Duplex phase particles of the Al–Fe phase coated with Al_4_C_3_ and Al_4_C_3_ cluster particles are two of the main nucleation particles in the carbon inoculated Fe-containing Mg-3%Al alloy. They can act as potent nuclei for α-Mg grains. The grain refining efficiency of the duplex phase particle is higher than that of the Al_4_C_3_ cluster particle due to the larger size of the duplex phase particle.

2. A thermodynamic model was established to reveal the formation mechanism of two kinds of particles by the change of Gibbs free energy. This model can explain and predict the in-situ formation trend of nucleation particles.

3. Thermodynamic model calculation results show that both duplex-phase particles and Al_4_C_3_ cluster particles can in-situ form spontaneously in a Mg-3%Al melt. The change of Gibbs free energy is negative from a magnitude of 10^9^ J/mol to 10^17^ J/mol. The free energy change caused by the same number of particles agglomerated to form Al_4_C_3_ cluster particles ranges from 10^8^ J/mol to 10^17^ J/mol. The adsorption process is more spontaneous than the cluster process, as the tiny Al_4_C_3_ particles prefer to form duplex phase particles rather than gathering to form Al_4_C_3_ cluster particles. The trend of forming stable duplex-phase particles becomes more and more obvious during the carbon inoculation process.

4. After adding Ca, all changes in Gibbs free energy, with different adsorptions of the Al_4_C_3_ layers, are negative. The addition of Ca can reduce the resistance of forming duplex phase particles and promote the formation of duplex-phase particles. Furthermore, the size of duplex-phase particles could increase by adsorbing more Al_4_C_3_ layers after the Ca addition.

## Figures and Tables

**Table 1:** List of the related parameters used in calculating the constitutional undercooling.

Parameter	Symbol	Value	Reference
Gradient of the solidus line	$m_{l}$-Al	−6.87	[34]
	$m_{l}$-Ca	−12.87	[34]
	$m_{l}$-Fe	−5.5	[34]
Equilibrium distribution coefficient	k-Al	0.37	[34]
	k-Ca	0.06	[34]
	k-Fe	0.054	[34]
